# Supplementary material for: Statistically consistent divide-and-conquer pipelines for phylogeny estimation using NJMerge
Source: Algorithms Mol Biol. 2019 Jul 19;14:14. doi: 10.1186/s13015-019-0151-x (PMC6642500; doi:10.1186/s13015-019-0151-x)
Supplement: Supplementary file 1 — Additional file 1. Detailed methods section, including software commands. 12 figures and 10 tables describing additional results, including those for exon-like datasets and those for analyses using log-det distance matrices. [file 13015_2019_151_MOESM1_ESM.pdf]

Statistically consistent divide-and-conquer pipelines for phylogeny estimation using NJMerge

Supplementary Materials

Erin K. Molloy and Tandy Warnow

April 20, 2019

Contents

List of Tables **1**

List of Figures **1**

References **26**

List of Tables

S1 INDELible Simulation Parameters . . . 2
S2 Simulated Dataset Properties . . . 4
S3 Gene Tree Median Branch Lengths . . . 4
S4 Running times for gene tree estimation (100-taxon datasets) . . . 7
S5 Running times for gene tree estimation (1000-taxon datasets) . . . 8
S6 Method Failures . . . 9
S7 Species tree error for NJMerge given AGID matrix . . . 22
S8 Species tree error for NJMerge given log-det distance matrix . . . 23
S9 Running times for NJMerge given AGID matrix . . . 24
S10 Running times for NJMerge given log-det distance matrix . . . 25

List of Figures

S1 Impact of distance matrix on NJMerge for exon-like datasets . . . 10
S2 Impact of estimated constraint trees on NJMerge for exon-like datasets . . . 11
S3 Comparison of species tree methods for exon-like datasets . . . 12
S4 Comparison ASTRAL and NJMerge given AGID matrix for exon-like datasets . . . 13
S5 Comparison of SVDquartets and NJMerge given AGID matrix) for exon-like datasets . . . 14
S6 Comparison RAxML of NJMerge given AGID matrix for exon-like datasets . . . 15
S7 Comparison ASTRAL and NJMerge given log-det distance matrix for intron-like datasets . . . 16
S8 Comparison of ASTRAL and NJMerge given log-det distance matrix for exon-like datasets . 17
S9 Comparison of SVDquartets and NJMerge given log-det distance matrix for intron-like datasets 18
S10 Comparison of SVDquartets and NJMerge given log-det distance matrix for exon-like datasets 19
S11 Comparison of RAxML and NJMerge given log-det distance matrix for intron-like datasets . 20
S12 Comparison of RAxML and NJMerge given log-det distance matrix for exon-like datasets . . 21

Supplementary Methods

Simulation Command

SimPhy [7] Version 1.0.2 was run as

```
simphy -rs 3000 -rl F:3000 -rg 1 -st F:[species tree height] \
       -si F:1 -sl F:[number of taxa] -sb F:0.0000001 -sp F:200000 \
       -hs LN:1.5,1 -hl LN:1.2,1 -hg LN:1.4,1 -su E:10000000 \
       -so F:1 -od 1 -v 3  -cs 293745 -o [output directory name]
```

where the number of taxa was 100 or 1000, the species tree height was 10,000,000 or 500,000 generations, and the effective population size was constant at 200,000.

INDELible Simulation

Like the simulation in [8], GTR+Γ model parameters (base frequencies, substitution rates, and alpha) were drawn from distributions. However, unlike the simulation in [8], we estimated separate distributions for exons, introns, and ultra-conserved elements (UCEs) from the Avian Phylogenomics Dataset [4]. GTR model parameters that were drawn from distributions given in Table S1. The α parameter for the Γ distribution was computed as follows. (1) For each gene, a random value x between 0 and 1 is drawn from a uniform distribution. (2) α was computed as $-log(x)/d$, where d is 4.2 for exons, 0.4 for introns, and 1.0 for UCEs. Because the mean of the uniform distribution is 0.5, the mean α is approximately 0.16 for exons, 1.73 for introns, and 0.69 for UCEs. We ran INDELible [3] Version 1.03 using custom Python scripts available on the Illinois Data Bank using the exon, intron, and UCE parameter distributions for genes 1-1000, 1001-2000, and 2001-3000, respectively. Only the exon-like and intron-like genes were used in this study due to limitations in computational resources.

Table S1: **INDELible Simulation Parameters.**

Sequence Type	GTR Base Frequencies	GTR Substitution Rates
Exon	Dirchlet(79,57,60,53)	Dirchlet(3,9,2,4,11,7)
Intron	Dirchlet(55,38,43,63)	Dirchlet(37,133,20,48,120,29)
UCE	Dirchlet(68,45,45,68)	Dirchlet(19,66,10,27,67,19)

Average Gene-Tree Internode Distance Matrix Commands

FastTree [10] Version 2.1.10 (SSE3) was run as

```
FastTree -nt -gtr -quiet -log fasttree-$gene.log \
     [input alignment fasta file] > [output FastTree-2 tree file]
```

ASTRID [15] Version 1.4 was run as

```
ASTRID -i [input gene tree list file] \
       -c [output distance matrix file] -o [temporary file]
```

LogDet Distance Matrix Command

PAUP* [14] 4a163 64-bit Centos was run as

```
echo  "ToNEXUS format=FASTA fromFile=[input alignment fasta file]
    toFile=[alignment nexus file]; exe [alignment nexus file]; DSet distance=logdet;
    SaveDist format=PHYLIP file=[output distance matrix file] triangle=both diagonal=yes;" |
    paup4a163_centos64 -n
```

Species Tree Commands

ASTRAL [16] Version 5.6.1 (i.e., ASTRAL-III) was run as

```
java java -Xms3200M -Xmx32000M ASTRAL/Astral/astral.5.6.1.jar \
     -i [input gene tree list file] -o [output ASTRAL-III tree file]
```

SVDquartets [1, 2] (PAUP* [14] Version 4a161/3) was run as

```
echo "exe [input alignment nexus file]; svd nthreads=16
    evalQuartets=all qfile=[output quartet file] qformat=qmc;
    savetrees file=[output SVDquartets tree file] format=newick;" |
    paup4a161_centos64 -n
```

PAUP* [14] Version 4a161 64-bit Centos was used for results obtained in [9], and PAUP* Version 4a163 64-bit Centos was used for results obtained using log-det distance matrix.

RAxML [11] Version 8.2.12 (with pThreads SSE3) was run as

```
raxmlHPC-PTHREADS-SSE3 -m GTRGAMMA -F -p [seed] \
    -n [output name] -s [input alignment file] -T 16
```

Note that the option `-j` (to write checkpoints) was included for the 1000-taxon datasets only.

NJ / NJMerge Commands

Neighbor Joining (FastME [5] Version 2.1.5) was run as

```
fastme -mN -i [input distance matrix file] -o [output tree file]
```

NJMerge was run as

```
python njmerge.py \
    -t [input constraint tree file 1] ... [input constraint tree file N] \
    -m [input internode distance matrix file] \
    -x [input rows to taxon name map file] \
    -o [output NJMerge tree file]
```

Tree Comparison Commands

The normalized symmetric difference was computed using Dendropy [13] Version 4.3.0 as

```
n1 = len(t1.internal_edges(exclude_seed_edge=True))
n2 = len(t2.internal_edges(exclude_seed_edge=True))
[fp, fn] = false_positives_and_negatives(t1, t2)
sd = float(fp + fn) / (n1 + n2)
```

where `t1` and `t2` are Dendropy tree objects. Note that the normalized symmetric difference equals the normalized Robinson-Foulds distance when both trees are fully resolved (i.e., binary). In this study, both true and estimated species trees as well as true gene trees were fully resolved; however, gene trees estimated using FastTree-2 could have polytomies.

Simulated Datasets

Table S2: **Simulated Dataset Properties.** Simulated datasets are described by the average discord between the species tree and the gene trees as well as the gene tree estimation error. Specifically, "Average Distance" is the normalized symmetric difference distance between the true species tree and the true gene tree, averaged across all 1000 genes in a replicate dataset. "Gene Tree Estimation Error" is the normalized symmetric difference between the true and the estimated gene trees, averaged across all 1000 genes in a replicate dataset. "Total Gene Tree Discord" is the normalized symmetric difference between the true species tree and the estimated gene tree, averaged across all 1000 genes in a replicate dataset. Values below are the mean (± standard deviation) across 20 replicates.

Number of Taxa	Sequence Type	Average Distance	Gene Tree Estimation Error	Total Gene Tree Discord
Moderate ILS (species tree height = 10M generations)				
100	exon	0.08 ± 0.02	0.38 ± 0.06	0.39 ± 0.06
100	intron	0.08 ± 0.02	0.26 ± 0.07	0.28 ± 0.06
1000	exon	0.10 ± 0.00	0.42 ± 0.04	0.43 ± 0.04
1000	intron	0.10 ± 0.00	0.30 ± 0.05	0.32 ± 0.05
Very High ILS (species tree height = 500K generations)				
100	exon	0.68 ± 0.02	0.57 ± 0.07	0.78 ± 0.03
100	intron	0.68 ± 0.02	0.43 ± 0.10	0.74 ± 0.03
1000	exon	0.69 ± 0.01	0.64 ± 0.05	0.81 ± 0.02
1000	intron	0.69 ± 0.01	0.51 ± 0.07	0.76 ± 0.03

Table S3: **Gene Tree Median Branch Lengths.** For the first replicate dataset in each model condition, we computed the median branch length per gene tree for the internal branches as well as the terminal branches, separately. Values below are the mean (± standard deviation) across 1000 gene trees.

Number of Taxa	Sequence Type	Internal Branch Lengths	Terminal Branch Lengths
Moderate ILS (species tree height = 10M generations)			
100	exon	0.0317 ± 0.0235	0.0588 ± 0.0436
100	intron	0.0317 ± 0.0229	0.0585 ± 0.0425
1000	exon	0.0255 ± 0.0184	0.0527 ± 0.0378
1000	intron	0.0260 ± 0.0189	0.0535 ± 0.0389
Very High ILS (species tree height = 500K generations)			
100	exon	0.0012 ± 0.0009	0.0072 ± 0.0051
100	intron	0.0012 ± 0.0010	0.0073 ± 0.0055
1000	exon	0.0010 ± 0.0007	0.0073 ± 0.0053
1000	intron	0.0009 ± 0.0007	0.0066 ± 0.0048

Approximation of Running Time for Gene Tree Estimation

We *re-estimated* gene trees in order to include the average time required for gene tree estimation for each replicate dataset (either 1000 exons or 1000 introns). Jobs were submitted using the qsub script below, where `$modl` is the model condition (e.g., `100tax-3000gen-10M`) and `$repl` is the replicate number (e.g., `08`). Technically, the script below estimates gene trees on gene numbered 1 to 1000 (i.e., 1000 exons), and a similar script was used to estimate gene trees numbered 1001 to 2000 (i.e., 1000 introns). This script will "launch serial tasks, up to [16] running concurently on a single node. Any number of tasks can be given, xargs will start the next task as soon as one of the currently running tasks finishes"; see `https://bluewaters.ncsa.illinois.edu/job-bundling` for more information.

```
#!/bin/bash
#PBS -A bakr
#PBS -j oe
#PBS -l nodes=1:ppn=16:xe
#PBS -l walltime=06:00:00
#PBS -l flags=commlocal:commtolerant

ts=$(date +%s)

source /opt/modules/default/init/bash

modl=$arg1
repl=$arg2

cd $PBS_O_WORKDIR

aprun -n 1 -N 1 -d 16 xargs -d '\n' -I cmd -P 16 /bin/bash -c 'cmd' <<EOF
./e_run_fasttree_for_timings.sh $modl $repl 1 62
./e_run_fasttree_for_timings.sh $modl $repl 63 125
./e_run_fasttree_for_timings.sh $modl $repl 126 187
./e_run_fasttree_for_timings.sh $modl $repl 188 250
./e_run_fasttree_for_timings.sh $modl $repl 251 312
./e_run_fasttree_for_timings.sh $modl $repl 313 375
./e_run_fasttree_for_timings.sh $modl $repl 376 437
./e_run_fasttree_for_timings.sh $modl $repl 438 500
./e_run_fasttree_for_timings.sh $modl $repl 401 562
./e_run_fasttree_for_timings.sh $modl $repl 563 625
./e_run_fasttree_for_timings.sh $modl $repl 626 687
./e_run_fasttree_for_timings.sh $modl $repl 688 750
./e_run_fasttree_for_timings.sh $modl $repl 751 812
./e_run_fasttree_for_timings.sh $modl $repl 813 875
./e_run_fasttree_for_timings.sh $modl $repl 876 937
./e_run_fasttree_for_timings.sh $modl $repl 938 1000
EOF

te=$(date +%s)
rt=$[te - ts]
echo "Estimated gene trees on $modl (exons) in $rt seconds"
```

Note that `./e_run_fasttree_for_timings.sh` simply runs FastTree-2 on genes numbered `$sgen` to `$egen`.

```
for gene in `seq -f "%04g" $sgen $egen`; do
    $fasttree -nt \
              -gtr \
              -quiet \
              -log fasttree-$gene.log \
              $gene.phy > fasttree-$gene.tre
done
```

Note that FastTree-2 was compiled using the following commands:

```
module unload darshan
module swap PrgEnv-cray PrgEnv-gnu
module swp gcc gcc/5.2.0  # Target: x86_64-suse-linux
gcc -DUSE_DOUBLE -O3 -finline-functions -funroll-loops \
    -Wall -o FastTreeDbl-no-darshan FastTree.c -lm
```

Table S4: **Running times for gene tree estimation (100-taxon datasets).** Running times (in seconds) for gene tree estimation is the time required to run FastTree-2 on datasets with 100 taxa and either 1000 exons or 1000 introns using a single Blue Waters compute node with 64 GB of memory and 16 floating-point cores. On average ($\pm$ standard deviation), gene tree estimation required 1080 ± 107 seconds (18 ± 2 minutes).

Species Tree Height	Replicate Number	Time (s) *for 1000 exons*	Time (s) *for 1000 introns*
10M	01	1075	1074
10M	02	884	946
10M	03	1166	1264
10M	04	1101	1095
10M	05	1217	1097
10M	06	1132	1121
10M	07	1189	1361
10M	08	1011	1042
10M	09	1234	1241
10M	10	1032	995
10M	11	1113	1153
10M	12	1084	1104
10M	13	1127	1130
10M	14	1091	1157
10M	15	1218	1235
10M	16	1106	1021
10M	17	1173	1252
10M	18	1111	1236
10M	19	1167	1175
10M	20	1060	1093
500K	01	913	874
500K	02	1172	1064
500K	03	1126	1149
500K	04	1025	1016
500K	05	1014	1015
500K	06	1000	1054
500K	07	927	893
500K	08	978	875
500K	09	1181	1197
500K	10	1093	1098
500K	11	1042	995
500K	12	987	904
500K	13	1082	1015
500K	14	964	941
500K	15	844	835
500K	16	1126	1126
500K	17	1180	1104
500K	18	1136	1113
500K	19	1107	1066
500K	20	1067	986

Table S5: **Running times for gene tree estimation (1000-taxon datasets).** Running time for gene tree estimation is the time required to run FastTree-2 on datasets with 1000 taxa and either 1000 exons or 1000 introns using a single Blue Waters compute node with 64 GB of memory and 16 floating-point cores. On average ($\pm$ standard deviation), gene tree estimation required 13018 ± 1169 seconds (217 ± 20 minutes).

Species Tree Height	Replicate Number	Time (s) *for 1000 exons*	Time (s) *for 1000 introns*
10M	01	13083	12295
10M	02	13310	13014
10M	03	10546	10948
10M	04	12544	11781
10M	05	13530	13673
10M	06	12430	10969
10M	07	14043	13735
10M	08	14588	15273
10M	09	13415	13045
10M	10	14336	13740
10M	11	14455	14214
10M	12	12596	11266
10M	13	13340	13056
10M	14	12159	11721
10M	15	14154	13084
10M	16	13247	12422
10M	17	13677	12949
10M	18	11600	11295
10M	19	13747	13045
10M	20	13940	13176
500K	01	11632	12232
500K	02	11380	10640
500K	03	13387	12627
500K	04	13938	13733
500K	05	12425	12280
500K	06	9460	9648
500K	07	13652	13085
500K	08	12464	11671
500K	09	13755	12320
500K	10	13912	13785
500K	11	14128	13534
500K	12	14416	14221
500K	13	12361	13029
500K	14	14402	14394
500K	15	13128	13920
500K	16	14148	13238
500K	17	14407	13344
500K	18	14279	13794
500K	19	12874	12264
500K	20	14283	13778

Supplementary Results

We often specify the input parameters when referring to Neighbor Joining (NJ) and NJMerge. For example, NJ(D_{AGID}) refers to NJ given the average gene tree internode distance (AGID) matrix as input, and NJMerge($\mathcal{T}_{RAX}, D_{AGID}$) refers to NJMerge given the constraint trees estimated using RAxML and the AGID matrix as input.

Distance matrices were created using two different approaches.

- D_{AGID} refers to the Average Gene tree Internode Distance matrix (as described in [6]) from estimated gene trees using ASTRID [15].
- D_{LD} refers to the logdet distance matrix (as described in [12]) and computed from concatenated alignment using PAUP* [14].

Constraint trees were created using four different approaches.

- $\mathcal{T}_{true}$ refers to constraint trees computed by restricting the true species tree to each subset of species.
- $\mathcal{T}_{AST}$ refers to constraint trees computed by running ASTRAL-III on each subset, i.e., on estimated gene trees restricted to subsets of species.
- $\mathcal{T}_{SVD}$ refers to constraint trees computed by running SVDquartets on each subset, i.e., on the concatenated alignment restricted to subsets of species.
- $\mathcal{T}_{RAX}$ refers to constraint trees computed by running RAxML on each subset, i.e., on the concatenated alignment restricted to subsets of species.

Table S6: **Method Failures.** Methods were run on 20 replicate datasets for each model condition with 1000 species, 1000 genes, two levels of ILS (species tree heights: 10M and 500K), and two sequence types (exon and intron). All four methods (ASTRAL-III, SVDquartets, RAxML, and NJMerge) failed on some datasets, as recorded below. ASTRAL-III failed due to running beyond the maximum wall-clock time of 48 hours; SVDquartets failed due to segmentation faults; RAxML failed due to running out of memory, and NJMerge failed due to being unable to find a legal siblinghood.

# of Taxa	# of Genes	Species Tree Height	Data Type	Method	Fraction of Replicates	Replicate Numbers
100	25	500K	exon	NJMerge($\mathcal{T}_{true}$, D_{LD})	1/20	10
100	25	500K	exon	NJMerge($\mathcal{T}_{RAX}$, D_{AGID})	1/20	16
100	25	500K	intron	NJMerge($\mathcal{T}_{true}$, D_{AGID})	1/20	6
1000	1000	10M	exon	SVDquartets	20/20	All
1000	1000	10M	exon	RAxML	3/20	2, 8, 17
1000	1000	10M	intron	NJMerge($\mathcal{T}_{AST}$, D_{LD})	1/20	20
1000	1000	10M	intron	SVDquartets	20/20	All
1000	1000	10M	intron	RAxML	20/20	All
1000	1000	500K	exon	ASTRAL-III	19/20	All except 15
1000	1000	500K	exon	NJMerge($\mathcal{T}_{true}$, D_{LD})	1/20	18
1000	1000	500K	exon	NJMerge($\mathcal{T}_{AST}$, D_{LD})	1/20	18
1000	1000	500K	exon	NJMerge($\mathcal{T}_{SVD}$, D_{LD})	2/20	14, 18
1000	1000	500K	exon	NJMerge($\mathcal{T}_{RAX}$, D_{LD})	2/20	14, 18
1000	1000	500K	exon	SVDquartets	20/20	All
1000	1000	500K	intron	ASTRAL-III	4/20	1, 5, 6, 20
1000	1000	500K	intron	NJMerge($\mathcal{T}_{SVD}, D_{LD}$)	1/20	6
1000	1000	500K	intron	SVDquartets	20/20	All
1000	1000	500K	intron	RAxML	19/20	All except 6

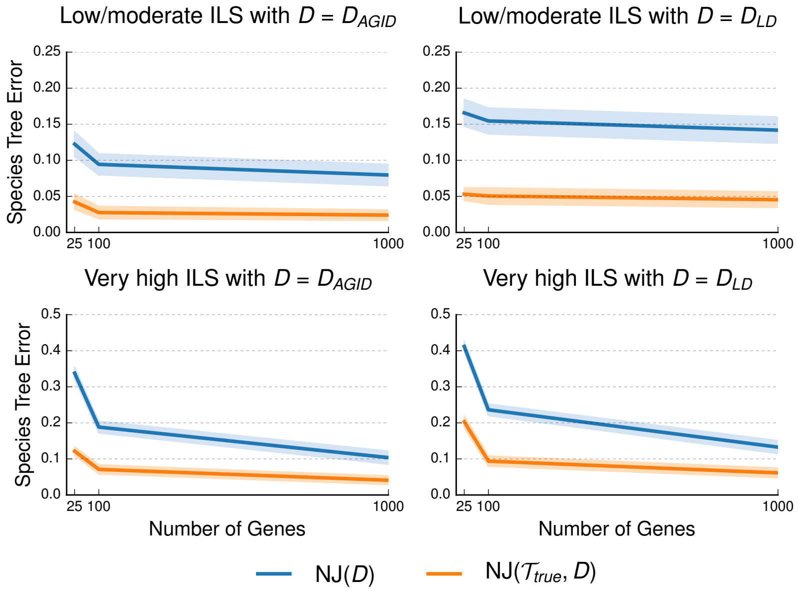

Figure S1: **Impact of distance matrix on NJMerge for 100-taxon, exon-like datasets.** Neighbor Joining (NJ) was run with two different distance matrices, and NJMerge was run with two different distance matrices and constraint trees that agreed with the true species tree (see the Performance Study section for more information on the notation). Datasets had two different levels of incomplete lineage sorting (ILS) and numbers of genes varying from 25 to 1000. Species tree estimation error is defined as the normalized Robinson-Foulds (RF) distance between true and estimated species trees. Lines represent the average over replicate datasets, and filled regions indicate the standard error.

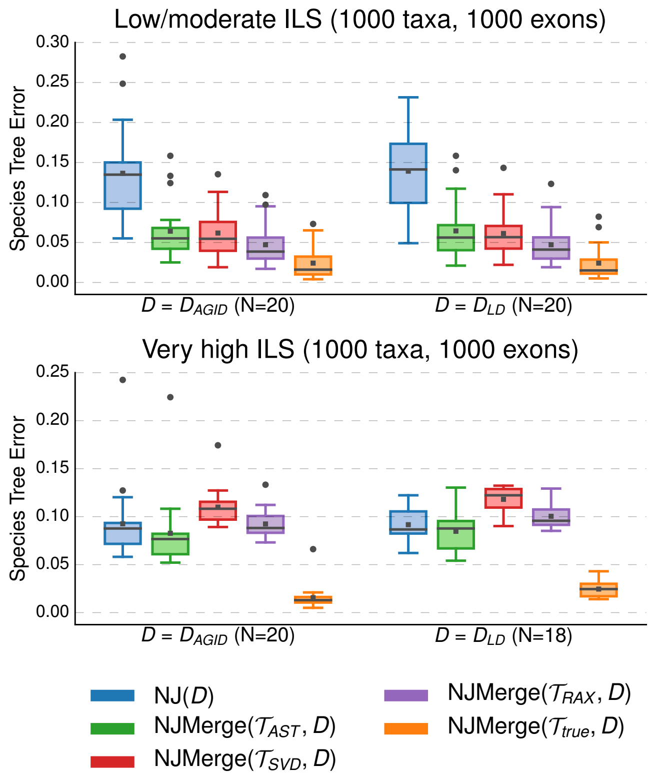

Figure S2: **Impact of constraint trees on NJMerge for 1000-taxon, exon-like datasets.** Neighbor Joining (NJ) was run with two different distance matrices, and NJMerge was run with two difference distance matrices and four different sets of constraint trees; see notation section above for details. Species tree estimation error is defined as the normalized Robinson-Foulds (RF) distance between true and estimated species trees. Note that gray bars represent medians, gray squares represent means, gray circles represent outliers, box plots are defined by quartiles (extending from the first to the third quartiles), and whiskers extend to plus/minus 1.5 times the interquartile distance (unless greater/less than the maximum/minimum value).

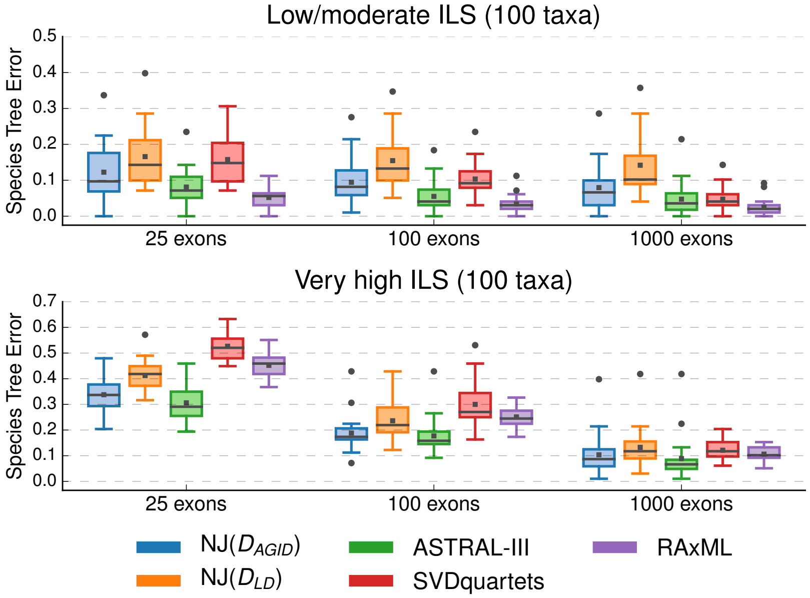

Figure S3: **Comparison of species tree methods for 100-taxon, exon-like datasets.** All methods were run on the full dataset (i.e., not subsets) with 100 species. Neighbor Joining (NJ) was run with two different distance matrices; see notation section above for details. Species tree estimation error is defined as the normalized Robinson-Foulds (RF) distance between true and estimated species trees. Note that gray bars represent medians, gray squares represent means, gray circles represent outliers, box plots are defined by quartiles (extending from the first to the third quartiles), and whiskers extend to plus/minus 1.5 times the interquartile distance (unless greater/less than the maximum/minimum value).

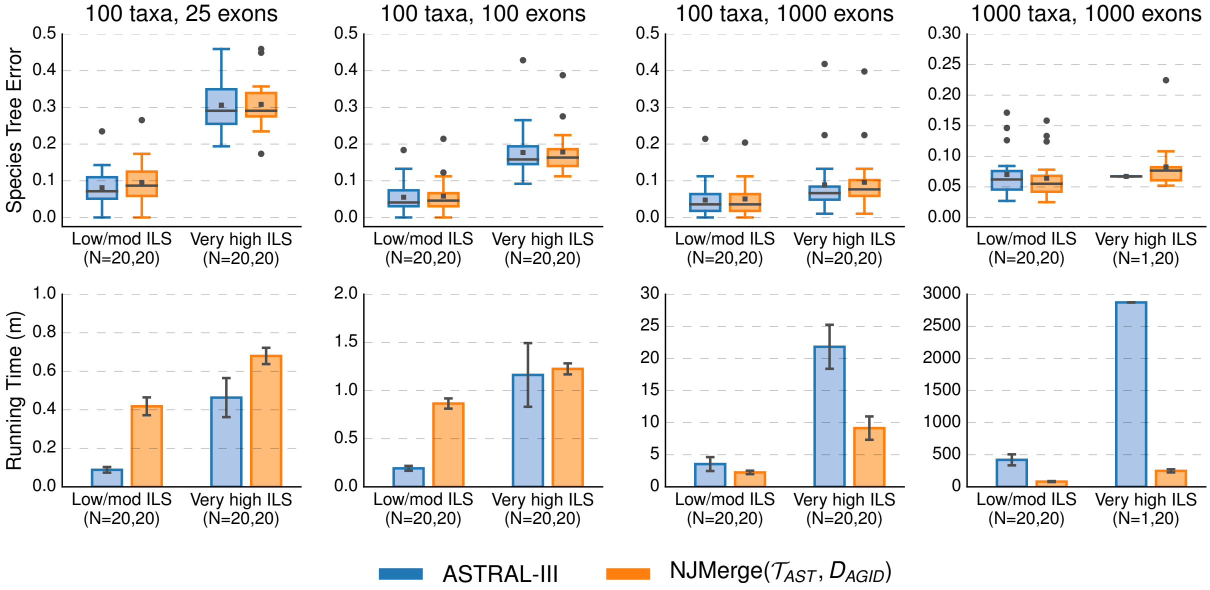

Figure S4: **Comparison of ASTRAL-III and NJMerge given ASTRAL-III constraint trees and AGID matrix for exon-like datasets.** Subplots on top row show species tree estimation error (defined as the normalized RF distance between true and estimated species trees); note that gray bars represent medians, gray squares represent means, gray circles represent outliers, box plots are defined by quartiles (extending from the first to the third quartiles), and whiskers extend to plus/minus 1.5 times the interquartile distance (unless greater/less than the maximum/minimum value). Subplots on bottom row show running time (in minutes); bars represent means and error bars represent standard deviations across replicate datasets. NJMerge running times is for computing the subset trees "in serial"; see Equation (1) in the main text for more information. The numbers of replicates on which the methods completed is shown on the x-axis, e.g., $N = X, Y$ indicates that ASTRAL-III completed on X out of 20 replicates and that NJMerge($\mathcal{T}_{AST}, D_{LD}$) completed on Y out of 20 replicates.

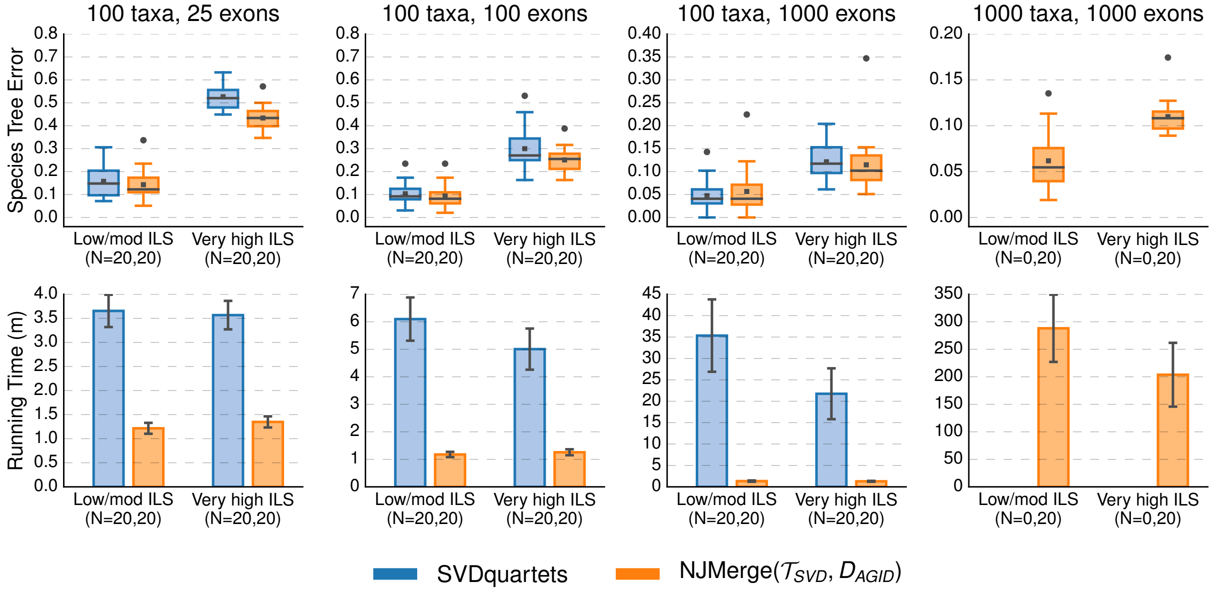

Figure S5: **Comparison of SVDquartets and NJMerge (given SVDquartets constraint trees and AGID matrix) for exon-like datasets.** Subplots on top row show species tree estimation error (defined as the normalized RF distance between true and estimated species trees); note that gray bars represent medians, gray squares represent means, gray circles represent outliers, box plots are defined by quartiles (extending from the first to the third quartiles), and whiskers extend to plus/minus 1.5 times the interquartile distance (unless greater/less than the maximum/minimum value). Subplots on bottom row show running time (in minutes); bars represent means and error bars represent standard deviations across replicate datasets. NJMerge running times is for computing the subset trees "in serial"; see Equation (1) in the main text for more information. The numbers of replicates on which the methods completed is shown on the x-axis, e.g., $N = X, Y$ indicates that SVDquartets completed on X out of 20 replicates and that NJMerge($\mathcal{T}_{SVD}, D_{LD}$) completed on Y out of 20 replicates.

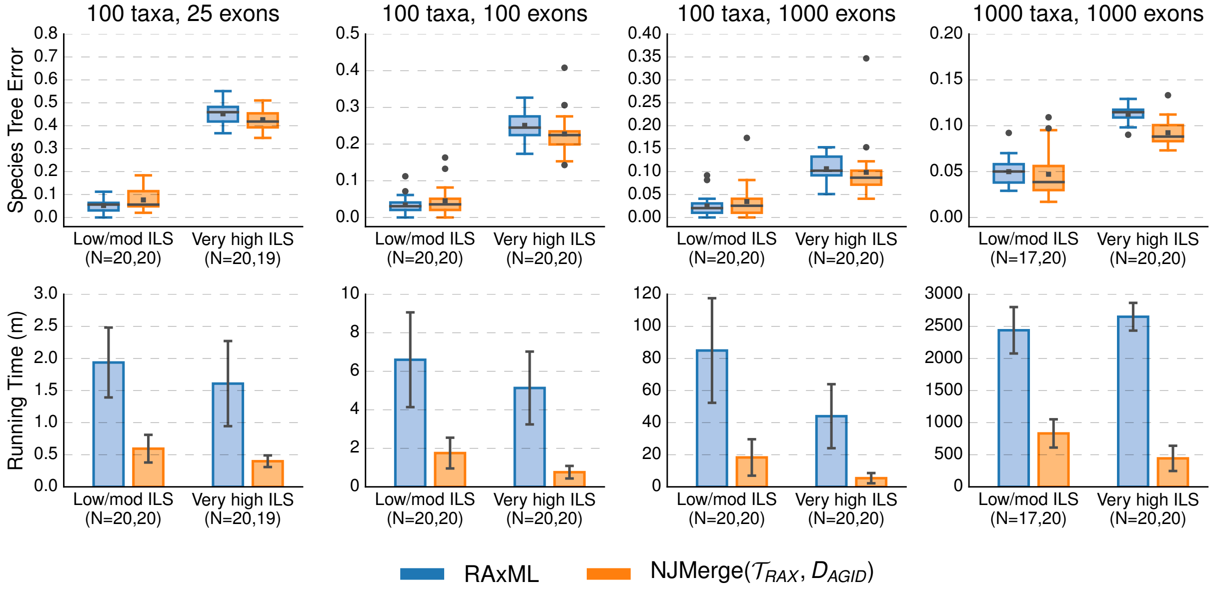

Figure S6: **Comparison of RAxML and NJMerge given RAxML constraint trees and AGID matrix for exon-like datasets.** Subplots on top row show species tree estimation error (defined as the normalized RF distance between true and estimated species trees); note that gray bars represent medians, gray squares represent means, gray circles represent outliers, box plots are defined by quartiles (extending from the first to the third quartiles), and whiskers extend to plus/minus 1.5 times the interquartile distance (unless greater/less than the maximum/minimum value). Subplots on bottom row show running time (in minutes); bars represent means and error bars represent standard deviations across replicate datasets. NJMerge running times is for computing the subset trees "in serial"; see Equation (1) in the main text for more information. The numbers of replicates on which the methods completed is shown on the x-axis, e.g., $N = X, Y$ indicates that RAxML completed on X out of 20 replicates and that NJMerge($\mathcal{T}_{RAX}, D_{LD}$) completed on Y out of 20 replicates.

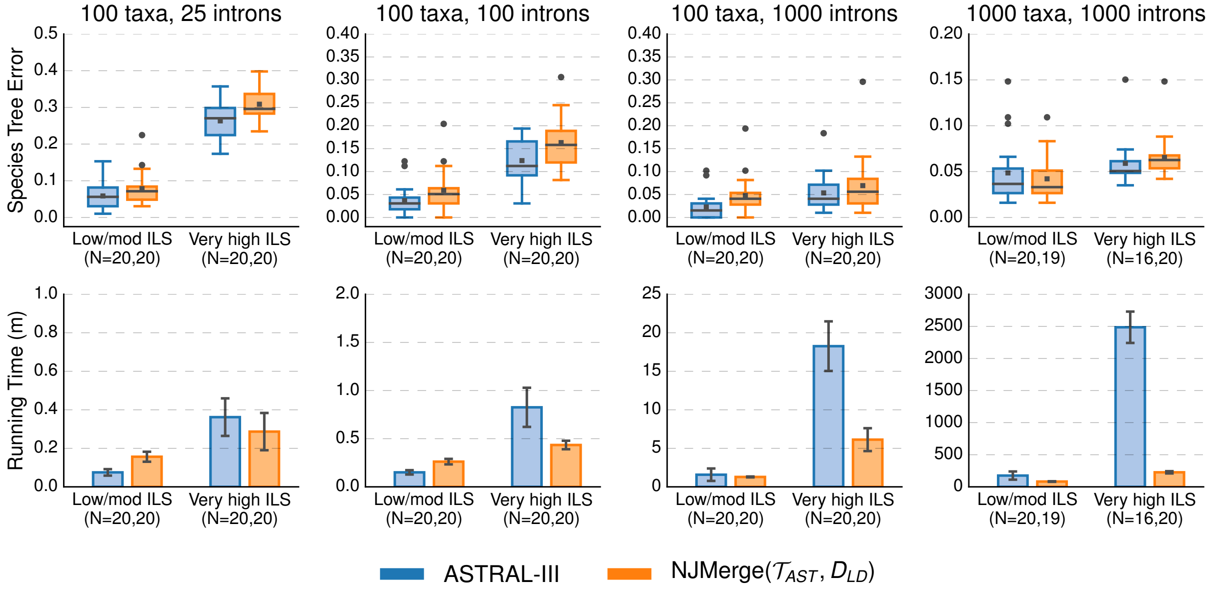

Figure S7: **Comparison of ASTAL-III and NJMerge given ASTRAL-III constraint trees and log-det distance matrix for intron-like datasets.** Subplots on top row show species tree estimation error (defined as the normalized RF distance between true and estimated species trees); note that gray bars represent medians, gray squares represent means, gray circles represent outliers, box plots are defined by quartiles (extending from the first to the third quartiles), and whiskers extend to plus/minus 1.5 times the interquartile distance (unless greater/less than the maximum/minimum value). Subplots on bottom row show running time (in minutes); bars represent means and error bars represent standard deviations across replicate datasets. NJMerge running times is for computing the subset trees "in serial"; see Equation (1) in the main text for more information. The numbers of replicates on which the methods completed is shown on the x-axis, e.g., $N = X, Y$ indicates that ASTRAL-III completed on X out of 20 replicates and that NJMerge($\mathcal{T}_{AST}, D_{LD}$) completed on Y out of 20 replicates.

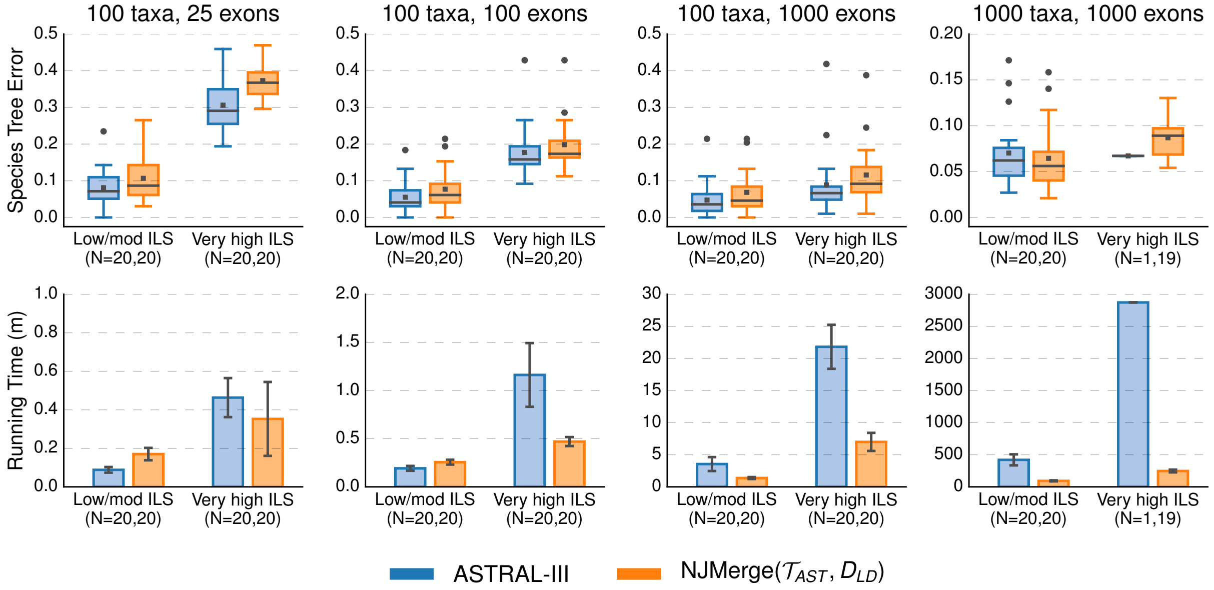

Figure S8: **Comparison of ASTRAL-III and NJMerge given ASTRAL-III constraint trees and log-det distance matrix for exon-like datasets.** Subplots on top row show species tree estimation error (defined as the normalized RF distance between true and estimated species trees); note that gray bars represent medians, gray squares represent means, gray circles represent outliers, box plots are defined by quartiles (extending from the first to the third quartiles), and whiskers extend to plus/minus 1.5 times the interquartile distance (unless greater/less than the maximum/minimum value). Subplots on bottom row show running time (in minutes); bars represent means and error bars represent standard deviations across replicate datasets. NJMerge running times is for computing the subset trees "in serial"; see Equation (1) in the main text for more information. The numbers of replicates on which the methods completed is shown on the x-axis, e.g., $N = X, Y$ indicates that ASTRAL-III completed on X out of 20 replicates and that NJMerge($\mathcal{T}_{AST}, D_{LD}$) completed on Y out of 20 replicates.

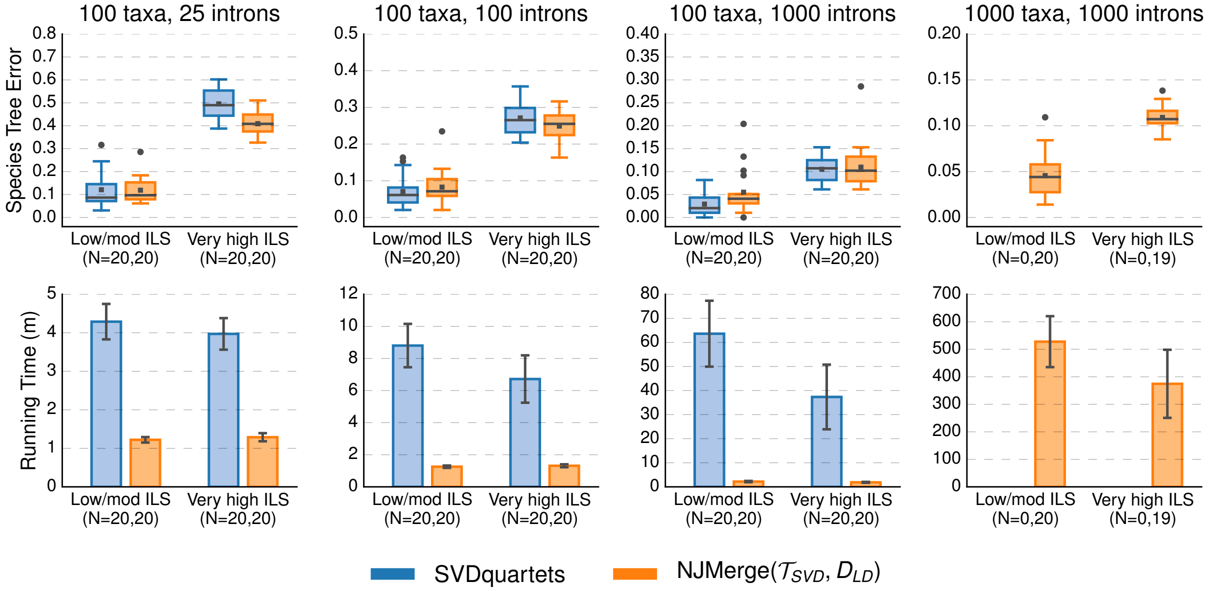

Figure S9: **Comparison of SVDquartets and NJMerge given SVDquartet constraint trees and log-det distance matrix for intron-like datasets.** Subplots on top row show species tree estimation error (defined as the normalized RF distance between true and estimated species trees); note that gray bars represent medians,, gray squares represent means, gray circles represent outliers, box plots are defined by quartiles (extending from the first to the third quartiles), and whiskers extend to plus/minus 1.5 times the interquartile distance (unless greater/less than the maximum/minimum value). Subplots on bottom row show running time (in minutes); bars represent means and error bars represent standard deviations across replicate datasets. NJMerge running times is for computing the subset trees "in serial"; see Equation (1) in the main text for more information. The numbers of replicates on which the methods completed is shown on the x-axis, e.g., $N = X, Y$ indicates that SVDquartets completed on X out of 20 replicates and that NJMerge($\mathcal{T}_{SVD}, D_{LD}$) completed on Y out of 20 replicates.

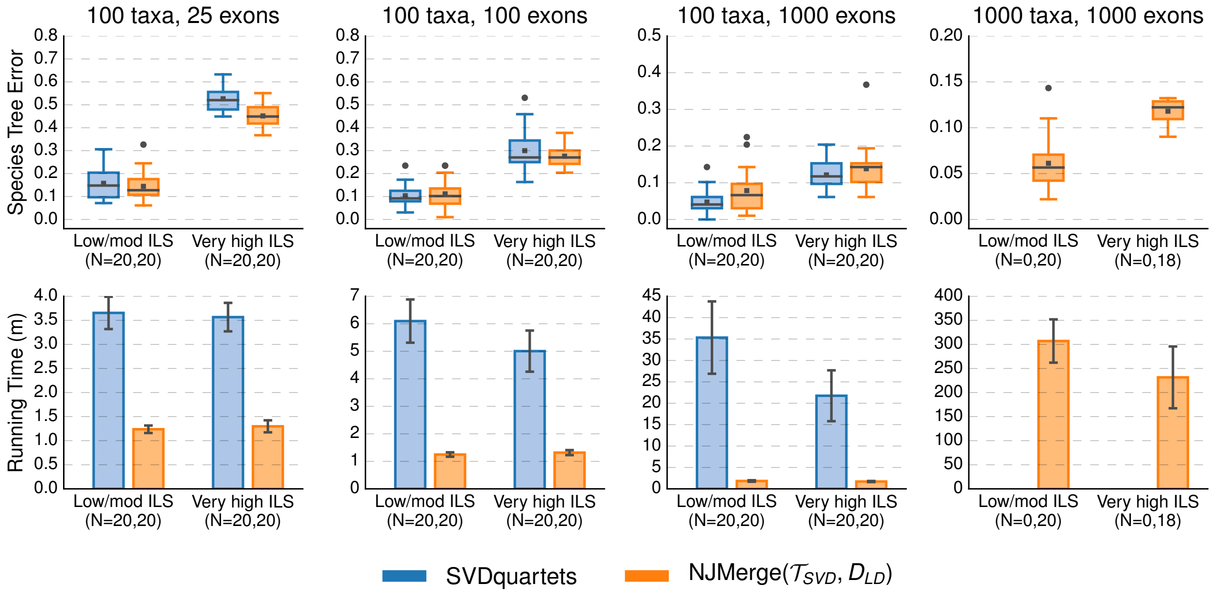

Figure S10: **Comparison of SVDquartets and NJMerge given log-det distance matrix for exon-like datasets.** Subplots on top row show species tree estimation error (defined as the normalized RF distance between true and estimated species trees); note that gray bars represent medians, gray squares represent means, gray circles represent outliers, box plots are defined by quartiles (extending from the first to the third quartiles), and whiskers extend to plus/minus 1.5 times the interquartile distance (unless greater/less than the maximum/minimum value). Subplots on bottom row show running time (in minutes); bars represent means and error bars represent standard deviations across replicate datasets. NJMerge running times is for computing the subset trees "in serial"; see Equation (1) in the main text for more information. The numbers of replicates on which the methods completed is shown on the x-axis, e.g., $N = X, Y$ indicates that SVDquartets completed on X out of 20 replicates and that NJMerge($\mathcal{T}_{SVD}, D_{LD}$) completed on Y out of 20 replicates.

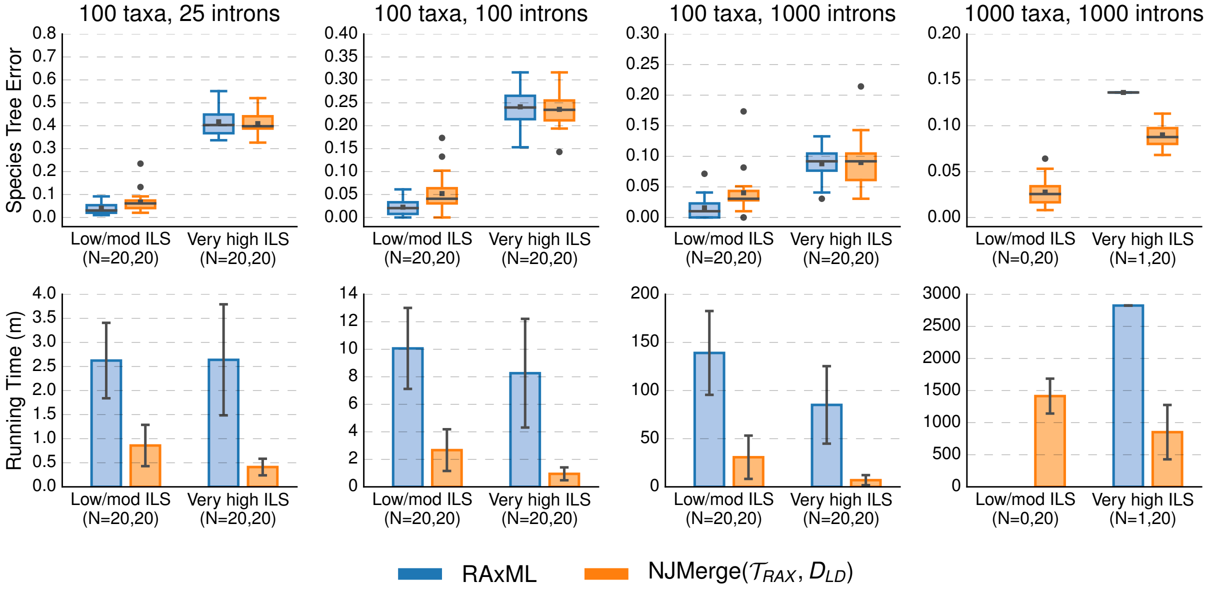

Figure S11: **Comparison of RAxML and NJMerge given RAxML constraint trees and log-det distance matrix for intron-like datasets.** Subplots on top row show species tree estimation error (defined as the normalized RF distance between true and estimated species trees); note that gray bars represent medians, gray squares represent means, gray circles represent outliers, box plots are defined by quartiles (extending from the first to the third quartiles), and whiskers extend to plus/minus 1.5 times the interquartile distance (unless greater/less than the maximum/minimum value). Subplots on bottom row show running time (in minutes); bars represent means and error bars represent standard deviations across replicate datasets. NJMerge running times is for computing the subset trees "in serial"; see Equation (1) in the main text for more information. The numbers of replicates on which the methods completed is shown on the x-axis, e.g., $N = X, Y$ indicates that RAxML completed on X out of 20 replicates and that NJMerge($\mathcal{T}_{RAX}, D_{LD}$) completed on Y out of 20 replicates.

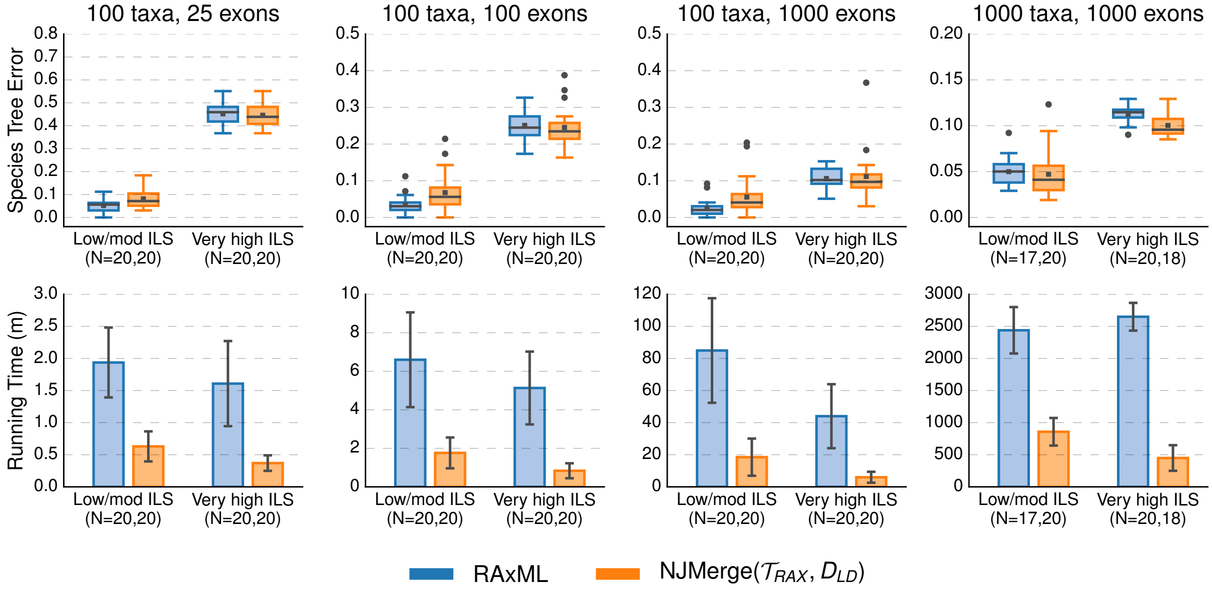

Figure S12: **Comparison of RAxML and NJMerge given RAxML constraint trees and log-det distance matrix for exon-like datasets.** Subplots on top row show species tree estimation error (defined as the normalized RF distance between true and estimated species trees); note that gray bars represent medians, gray squares represent means, gray circles represent outliers, box plots are defined by quartiles (extending from the first to the third quartiles), and whiskers extend to plus/minus 1.5 times the interquartile distance (unless greater/less than the maximum/minimum value). Subplots on bottom row show running time (in minutes); bars represent means and error bars represent standard deviations across replicate datasets. NJMerge running times is for computing the subset trees "in serial"; see Equation (1) in the main text for more information. The numbers of replicates on which the methods completed is shown on the x-axis, e.g., $N = X, Y$ indicates that RAxML completed on X out of 20 replicates and that NJMerge($\mathcal{T}_{RAX}, D_{LD}$) completed on Y out of 20 replicates.

Table S7: **Species tree error for NJMerge given AGID matrix.** Each species tree estimation method (ASTRAL-III, SVDquartets, or RAML) was run on the full dataset or on subsets in order to build constraint trees for NJMerge. We report the average (± standard deviation) species tree estimation error for *1)* the tree produced by running species tree method M on the full set of species (all 100 or all 1000 taxa), *2)* the tree produced by running species tree method M on subsets of species to produce $\mathcal{T}_M$, *3)* the tree produced by running NJ(D_{AGID}), and *4)* running NJMerge($\mathcal{T}_M, D_{AGID}$). Species tree estimation error (defined as normalized RF distance between the true and the estimated species tree) was averaged across 20 replicate datasets, unless the number of replicate datasets is otherwise noted in parentheses. When methods were run on subsets, species tree estimation error was averaged across all subsets and all replicate datasets. Note that the number of taxa in the subset trees was less than 30 for the 100-taxon datasets and less than 120 for the 1000-taxon datasets.

Number of Taxa	Number of Genes	Species Tree Height	Data Type	M on full dataset	M on subsets	NJ(D_{AGID})	NJMerge($\mathcal{T}_M, D_{AGID}$)
M = ASTRAL-III							
100	25	10M	exon	0.08	0.07	0.12	0.10
100	25	10M	intron	0.06	0.05	0.07	0.06
100	25	500K	exon	0.31	0.25	0.34	0.31
100	25	500K	intron	0.26	0.20	0.30	0.26
100	100	10M	exon	0.06	0.04	0.09	0.06
100	100	10M	intron	0.04	0.03	0.05	0.04
100	100	500K	exon	0.18	0.12	0.19	0.18
100	100	500K	intron	0.12	0.09	0.14	0.13
100	1000	10M	exon	0.05	0.03	0.08	0.05
100	1000	10M	intron	0.02	0.02	0.04	0.03
100	1000	500K	exon	0.09	0.06	0.10	0.10
100	1000	500K	intron	0.05	0.04	0.06	0.06
1000	1000	10M	exon	0.07	0.04	0.14	0.06
1000	1000	10M	intron	0.05	0.03	0.11	0.05
1000	1000	500K	exon	0.07 (1)	0.07	0.09	0.08
1000	1000	500K	intron	0.06 (16)	0.05	0.07	0.06
M = SVDquartets							
100	25	10M	exon	0.16	0.12	0.12	0.14
100	25	10M	intron	0.12	0.09	0.07	0.10
100	25	500K	exon	0.53	0.38	0.34	0.43
100	25	500K	intron	0.50	0.34	0.30	0.39
100	100	10M	exon	0.10	0.08	0.09	0.09
100	100	10M	intron	0.07	0.06	0.05	0.07
100	100	500K	exon	0.30	0.21	0.19	0.25
100	100	500K	intron	0.27	0.20	0.14	0.23
100	1000	10M	exon	0.05	0.04	0.08	0.06
100	1000	10M	intron	0.03	0.03	0.04	0.03
100	1000	500K	exon	0.12	0.08	0.10	0.11
100	1000	500K	intron	0.11	0.08	0.06	0.10
1000	1000	10M	exon	NA (0)	0.04	0.14	0.06
1000	1000	10M	intron	NA (0)	0.03	0.11	0.05
1000	1000	500K	exon	NA (0)	0.10	0.09	0.11
1000	1000	500K	intron	NA (0)	0.09	0.07	0.10
M = RAxML							
100	25	10M	exon	0.05	0.04	0.12	0.08
100	25	10M	intron	0.04	0.03	0.07	0.05
100	25	500K	exon	0.45	0.38	0.34	0.43 (19)
100	25	500K	intron	0.42	0.34	0.30	0.39
100	100	10M	exon	0.03	0.02	0.09	0.05
100	100	10M	intron	0.02	0.02	0.05	0.03
100	100	500K	exon	0.25	0.18	0.19	0.23
100	100	500K	intron	0.24	0.18	0.14	0.21
100	1000	10M	exon	0.02	0.01	0.08	0.03
100	1000	10M	intron	0.02	0.01	0.04	0.02
100	1000	500K	exon	0.11	0.06	0.10	0.10
100	1000	500K	intron	0.09	0.06	0.06	0.08
1000	1000	10M	exon	0.05 (17)	0.02	0.14	0.05
1000	1000	10M	intron	NA (0)	0.01	0.11	0.03
1000	1000	500K	exon	0.11	0.08	0.09	0.09
1000	1000	500K	intron	0.14 (1)	0.08	0.07	0.08

Table S8: **Species tree error for NJMerge given log-det distance matrix.** Each species tree estimation method (ASTRAL-III, SVDquartets, or RAML) was run on the full dataset or on subsets in order to build constraint trees for NJMerge. We report the average (± standard deviation) species tree estimation error for *1)* the tree produced by running species tree method M on the full set of species (all 100 or all 1000 taxa), *2)* the tree produced by running species tree method M on subsets of species to produce $\mathcal{T}_M$, *3)* the tree produced by running NJ(D_{LD}), and *4)* running NJMerge($\mathcal{T}_M, D_{LD}$). Species tree estimation error (defined as normalized RF distance between the true and the estimated species tree) was averaged across 20 replicate datasets, unless the number of replicate datasets is otherwise noted in parentheses. When methods were run on subsets, species tree estimation error was averaged across all subsets and all replicate datasets. Note that the number of taxa in the subset trees was less than 30 for the 100-taxon datasets and less than 120 for the 1000-taxon datasets.

Number of Taxa	Number of Genes	Species Tree Height	Data Type	M on full dataset	M on subsets	NJ(D_{LD})	NJMerge($\mathcal{T}_M, D_{LD}$)
M = ASTRAL-III							
100	25	10M	exon	0.08	0.06	0.17	0.11
100	25	10M	intron	0.06	0.04	0.14	0.08
100	25	500K	exon	0.31	0.26	0.41	0.37
100	25	500K	intron	0.26	0.19	0.36	0.31
100	100	10M	exon	0.06	0.04	0.15	0.08
100	100	10M	intron	0.04	0.03	0.12	0.06
100	100	500K	exon	0.18	0.13	0.24	0.20
100	100	500K	intron	0.12	0.10	0.21	0.16
100	1000	10M	exon	0.05	0.03	0.14	0.07
100	1000	10M	intron	0.02	0.02	0.11	0.05
100	1000	500K	exon	0.09	0.06	0.13	0.12
100	1000	500K	intron	0.05	0.04	0.09	0.07
1000	1000	10M	exon	0.07	0.04	0.14	0.06
1000	1000	10M	intron	0.05	0.03	0.11	0.04 (19)
1000	1000	500K	exon	0.07 (1)	0.07	0.10	0.09 (19)
1000	1000	500K	intron	0.06 (16)	0.05	0.07	0.07
M = SVDquartets							
100	25	10M	exon	0.16	0.11	0.17	0.14
100	25	10M	intron	0.12	0.09	0.14	0.12
100	25	500K	exon	0.53	0.36	0.41	0.45
100	25	500K	intron	0.50	0.32	0.36	0.41
100	100	10M	exon	0.10	0.07	0.15	0.11
100	100	10M	intron	0.07	0.05	0.12	0.08
100	100	500K	exon	0.30	0.22	0.24	0.28
100	100	500K	intron	0.27	0.19	0.21	0.25
100	1000	10M	exon	0.05	0.04	0.14	0.08
100	1000	10M	intron	0.03	0.03	0.11	0.06
100	1000	500K	exon	0.12	0.09	0.13	0.14
100	1000	500K	intron	0.11	0.08	0.09	0.11
1000	1000	10M	exon	NA (0)	0.04	0.14	0.06
1000	1000	10M	intron	NA (0)	0.03	0.11	0.05
1000	1000	500K	exon	NA (0)	0.10	0.10	0.12 (18)
1000	1000	500K	intron	NA (0)	0.09	0.07	0.11 (19)
M = RAxML							
100	25	10M	exon	0.05	0.04	0.17	0.08
100	25	10M	intron	0.04	0.03	0.14	0.07
100	25	500K	exon	0.45	0.34	0.41	0.45
100	25	500K	intron	0.42	0.32	0.36	0.41
100	100	10M	exon	0.03	0.02	0.15	0.07
100	100	10M	intron	0.02	0.02	0.12	0.05
100	100	500K	exon	0.25	0.19	0.24	0.25
100	100	500K	intron	0.24	0.18	0.21	0.24
100	1000	10M	exon	0.02	0.01	0.14	0.06
100	1000	10M	intron	0.02	0.01	0.11	0.04
100	1000	500K	exon	0.11	0.06	0.13	0.11
100	1000	500K	intron	0.09	0.06	0.09	0.09
1000	1000	10M	exon	0.05 (17)	0.02	0.14	0.05
1000	1000	10M	intron	NA (0)	0.01	0.11	0.03
1000	1000	500K	exon	0.11	0.08	0.10	0.10 (18)
1000	1000	500K	intron	0.14 (1)	0.07	0.07	0.09

Table S9: **Running times for NJMerge given AGID matrix.** Each species tree estimation method M was run on the full dataset (all 100 or all 1000 taxa) or on subsets in order to build a set $\mathcal{T}_M$ of constraint trees for NJMerge. We report the average running time ($\pm$ the standard deviation) in **seconds** across 20 replicate datasets, unless the number of replicate datasets is otherwise noted in parentheses. When methods were run on subsets, the time was measured per subset, and then average was taken across all subsets for all replicate datasets. Note that the 100-taxon datasets were decomposed into 4-6 subsets with a maximum subset size of 30 taxa and that the 1000-taxon datasets were decomposed into 10-15 subsets with a maximum subset size of 120 taxa.

Number of Taxa	Number of Genes	Species Tree Height	Data Type	M on full dataset	M on subsets	NJMerge($\mathcal{T}_M, D_{AGID}$)
M = *ASTRAL-III*						
100	25	10M	exon	5 ± 1	4 ± 1	5 ± 2
100	25	10M	intron	5 ± 1	4 ± 1	5 ± 1
100	25	500K	exon	28 ± 6	8 ± 2	5 ± 1
100	25	500K	intron	22 ± 6	7 ± 2	6 ± 2
100	100	10M	exon	12 ± 2	10 ± 2	5 ± 1
100	100	10M	intron	9 ± 1	9 ± 2	5 ± 1
100	100	500K	exon	70 ± 20	15 ± 3	5 ± 1
100	100	500K	intron	50 ± 12	14 ± 3	5 ± 1
100	1000	10M	exon	213 ± 65	28 ± 10	5 ± 1
100	1000	10M	intron	95 ± 48	21 ± 5	5 ± 1
100	1000	500K	exon	1309 ± 206	121 ± 56	5 ± 1
100	1000	500K	intron	1096 ± 193	103 ± 57	5 ± 1
1000	1000	10M	exon	25231 ± 5154	239 ± 119	1939 ± 66
1000	1000	10M	intron	10545 ± 3823	126 ± 68	1939 ± 74
1000	1000	500K	exon	172346 ± 0 (1)	1073 ± 529	1950 ± 283
1000	1000	500K	intron	149146 ± 14657 (16)	907 ± 394	1879 ± 24
M = *SVDquartets*						
100	25	10M	exon	219 ± 20	15 ± 5	6 ± 2
100	25	10M	intron	257 ± 28	15 ± 5	5 ± 2
100	25	500K	exon	214 ± 18	17 ± 5	9 ± 6
100	25	500K	intron	238 ± 25	15 ± 5	8 ± 4
100	100	10M	exon	366 ± 47	14 ± 5	5 ± 1
100	100	10M	intron	528 ± 81	14 ± 5	6 ± 2
100	100	500K	exon	300 ± 45	15 ± 6	6 ± 2
100	100	500K	intron	403 ± 89	15 ± 6	6 ± 2
100	1000	10M	exon	2120 ± 507	16 ± 7	5 ± 2
100	1000	10M	intron	3817 ± 821	19 ± 9	5 ± 2
100	1000	500K	exon	1305 ± 356	16 ± 6	5 ± 2
100	1000	500K	intron	2240 ± 806	16 ± 6	5 ± 2
1000	1000	10M	exon	$NA \pm NA$ (0)	1238 ± 1142	2005 ± 124
1000	1000	10M	intron	$NA \pm NA$ (0)	2219 ± 2019	1999 ± 184
1000	1000	500K	exon	$NA \pm NA$ (0)	839 ± 803	2057 ± 178
1000	1000	500K	intron	$NA \pm NA$ (0)	1550 ± 1615	1975 ± 76
M = *RAxML*						
100	25	10M	exon	116 ± 33	7 ± 5	5 ± 2
100	25	10M	intron	157 ± 47	10 ± 7	5 ± 2
100	25	500K	exon	96 ± 40	4 ± 3	8 ± 2 (19)
100	25	500K	intron	158 ± 69	4 ± 3	8 ± 3
100	100	10M	exon	396 ± 148	22 ± 16	5 ± 2
100	100	10M	intron	604 ± 177	32 ± 25	5 ± 1
100	100	500K	exon	308 ± 113	9 ± 7	6 ± 2
100	100	500K	intron	496 ± 237	10 ± 9	5 ± 2
100	1000	10M	exon	5097 ± 1955	238 ± 202	5 ± 1
100	1000	10M	intron	8343 ± 2611	426 ± 401	5 ± 1
100	1000	500K	exon	2641 ± 1196	71 ± 60	5 ± 1
100	1000	500K	intron	5106 ± 2414	83 ± 99	5 ± 1
1000	1000	10M	exon	146329 ± 21692 (17)	3887 ± 2023	2055 ± 165
1000	1000	10M	intron	$NA \pm NA$ (0)	6496 ± 3226	2010 ± 174
1000	1000	500K	exon	158973 ± 12955	2037 ± 1554	2006 ± 179
1000	1000	500K	intron	169440 ± 0 (1)	3976 ± 3203	1933 ± 86

Table S10: **Running times for NJMerge given log-det distance matrix.** Each species tree estimation method M was run on the full dataset (all 100 or all 1000 taxa) or on subsets in order to build a set $\mathcal{T}_M$ of constraint trees for NJMerge. We report the average running time ($\pm$ the standard deviation) in **seconds** across 20 replicate datasets, unless the number of replicate datasets is otherwise noted in parentheses. When methods were run on subsets, the time was measured per subset, and then average was taken across all subsets for all replicate datasets. Note that the 100-taxon datasets were decomposed into 4-6 subsets with a maximum subset size of 30 taxa and that the 1000-taxon datasets were decomposed into 10-14 subsets with a maximum subset size of 120 taxa.

Number of Taxa	Number of Genes	Species Tree Height	Data Type	M on full dataset	M on subsets	NJMerge($\mathcal{T}_M$, D_{LD})
M = *ASTRAL-III*						
100	25	10M	exon	5 ± 1	1 ± 0	6 ± 2
100	25	10M	intron	5 ± 1	1 ± 0	5 ± 1
100	25	500K	exon	28 ± 6	2 ± 1	13 ± 11
100	25	500K	intron	22 ± 6	2 ± 0	9 ± 6
100	100	10M	exon	12 ± 2	2 ± 0	5 ± 1
100	100	10M	intron	9 ± 1	1 ± 0	5 ± 1
100	100	500K	exon	70 ± 20	4 ± 2	6 ± 2
100	100	500K	intron	50 ± 12	4 ± 1	6 ± 1
100	1000	10M	exon	213 ± 65	10 ± 5	5 ± 2
100	1000	10M	intron	95 ± 48	6 ± 2	5 ± 1
100	1000	500K	exon	1309 ± 206	86 ± 44	5 ± 1
100	1000	500K	intron	1096 ± 193	73 ± 40	5 ± 1
1000	1000	10M	exon	25231 ± 5154	166 ± 80	2117 ± 309
1000	1000	10M	intron	10545 ± 3823	75 ± 45	2004 ± 123 (19)
1000	1000	500K	exon	172346 ± 0 (1)	945 ± 482	2126 ± 343 (19)
1000	1000	500K	intron	149146 ± 14657 (16)	773 ± 364	1991 ± 128
M = *SVDquartets*						
100	25	10M	exon	219 ± 20	16 ± 5	5 ± 2
100	25	10M	intron	257 ± 28	15 ± 5	5 ± 2
100	25	500K	exon	214 ± 18	15 ± 6	7 ± 3
100	25	500K	intron	238 ± 25	15 ± 5	7 ± 3
100	100	10M	exon	366 ± 47	15 ± 5	5 ± 1
100	100	10M	intron	528 ± 81	14 ± 5	5 ± 1
100	100	500K	exon	300 ± 45	16 ± 5	6 ± 2
100	100	500K	intron	403 ± 89	15 ± 5	6 ± 2
100	1000	10M	exon	2120 ± 507	16 ± 7	5 ± 1
100	1000	10M	intron	3817 ± 821	18 ± 9	5 ± 1
100	1000	500K	exon	1305 ± 356	16 ± 6	5 ± 1
100	1000	500K	intron	2240 ± 806	16 ± 6	5 ± 1
1000	1000	10M	exon	$NA \pm NA$ (0)	1225 ± 1044	2064 ± 324
1000	1000	10M	intron	$NA \pm NA$ (0)	2288 ± 2003	2022 ± 202
1000	1000	500K	exon	$NA \pm NA$ (0)	858 ± 845	2283 ± 457 (18)
1000	1000	500K	intron	$NA \pm NA$ (0)	1459 ± 1514	2081 ± 216 (19)
M = *RAxML*						
100	25	10M	exon	116 ± 33	7 ± 5	5 ± 1
100	25	10M	intron	157 ± 47	10 ± 8	5 ± 1
100	25	500K	exon	96 ± 40	3 ± 2	7 ± 4
100	25	500K	intron	158 ± 69	4 ± 4	6 ± 1
100	100	10M	exon	396 ± 148	22 ± 15	5 ± 1
100	100	10M	intron	604 ± 177	32 ± 25	5 ± 1
100	100	500K	exon	308 ± 113	9 ± 7	6 ± 1
100	100	500K	intron	496 ± 237	11 ± 10	6 ± 2
100	1000	10M	exon	5097 ± 1955	231 ± 201	5 ± 2
100	1000	10M	intron	8343 ± 2611	390 ± 366	5 ± 2
100	1000	500K	exon	2641 ± 1196	73 ± 63	5 ± 2
100	1000	500K	intron	5106 ± 2414	84 ± 96	5 ± 2
1000	1000	10M	exon	146329 ± 21692 (17)	3935 ± 1945	2200 ± 485
1000	1000	10M	intron	$NA \pm NA$ (0)	6697 ± 3093	2070 ± 232
1000	1000	500K	exon	158973 ± 12955	2022 ± 1463	2076 ± 200 (18)
1000	1000	500K	intron	169440 ± 0 (1)	3905 ± 3174	2259 ± 1164

References

[1] J. Chifman and L. Kubatko. Quartet Inference from SNP Data Under the Coalescent Model. *Bioinformatics*, 30(23):3317–3324, 2014.

[2] J. Chifman and L. Kubatko. Identifiability of the unrooted species tree topology under the coalescent model with time-reversible substitution processes, site-specific rate variation, and invariable sites. *Journal of Theoretical Biology*, 374:35–47, 2015.

[3] W. Fletcher and Z. Yang. INDELible: A Flexible Simulator of Biological Sequence Evolution. *Molecular Biology and Evolution*, 26(8):1879–1888, 2009.

[4] E. D. Jarvis, S. Mirarab, et al. Whole-genome analyses resolve early branches in the tree of life of modern birds. *Science*, 346(6215):1320–1331, 2014.

[5] V. Lefort, R. Desper, and O. Gascuel. FastME 2.0: A Comprehensive, Accurate, and Fast Distance-Based Phylogeny Inference Program. *Molecular Biology and Evolution*, 32(10):2798–2800, 2015.

[6] L. Liu and L. Yu. Estimating Species Trees from Unrooted Gene Trees. *Systematic Biology*, 60(5):661–667, 2011.

[7] D. Mallo, L. De Oliveira Martins, and D. Posada. SimPhy : Phylogenomic Simulation of Gene, Locus, and Species Trees. *Systematic Biology*, 65(2):334–344, 2016.

[8] S. Mirarab and T. Warnow. ASTRAL-II: coalescent-based species tree estimation with many hundreds of taxa and thousands of genes. *Bioinformatics*, 31(12):i44–i52, 2015.

[9] E. K. Molloy and T. Warnow. NJMerge: A Generic Technique for Scaling Phylogeny Estimation Methods and Its Application to Species Trees. In M. Blanchette and A. Ouangraoua, editors, *Comparative Genomics. RECOMB-CG 2018. Lecture Notes in Computer Science*, volume 11183. Springer, Cham, 2018.

[10] M. N. Price, P. S. Dehal, and A. P. Arkin. FastTree 2 - Approximately Maximum-Likelihood Trees for Large Alignments. *PLOS ONE*, 5(3):1–10, 2010.

[11] A. Stamatakis. RAxML version 8: a tool for phylogenetic analysis and post-analysis of large phylogenies. *Bioinformatics*, 30(9):1312–1313, 2014.

[12] M. Steel. Recovering a tree from the leaf colourations it generates under a Markov model. *Applied Mathematics Letters*, 7(2):19–24, 1994.

[13] J. Sukumaran and M. T. Holder. DendroPy: a Python library for phylogenetic computing. *Bioinformatics*, 26(12):1569–1571, 2010.

[14] D. L. Swofford. PAUP* (*Phylogenetic Analysis Using PAUP), Versions 4a161 and 4a163, 2018. http://phylosolutions.com/paup-test/.

[15] P. Vachaspati and T. Warnow. ASTRID: Accurate Species TRees from Internode Distances. *BMC Genomics*, 16(10):S3, 2015.

[16] C. Zhang, M. Rabiee, E. Sayyari, and S. Mirarab. ASTRAL-III: polynomial time species tree reconstruction from partially resolved gene trees. *BMC Bioinformatics*, 19(6):153, 2018.
